# Comparison of the Effectiveness and Environmental Impact of Selected Methods for the Determination of Fatty Acids in Milk Samples

**DOI:** 10.3390/molecules27238242

**Published:** 2022-11-25

**Authors:** Izabela Narloch, Grażyna Wejnerowska

**Affiliations:** Department of Food Analysis and Environmental Protection, Faculty of Chemical Technology and Engineering, UTP University of Science and Technology, 3 Seminaryjna Street, 85-326 Bydgoszcz, Poland

**Keywords:** environmental assessment tools, fatty acids, greenness, gas chromatography, milk fat

## Abstract

Determination of the fatty acid profile in milk samples is one of the most important in food analysis. There are many methodologies for FA determination. The conventional procedure for determining the FA composition of milk is isolation of fat or indirect methylation, trans-methylation, extraction of fatty acids, and analysis by gas chromatography. In this study, eight methods based on alkaline methylation were compared for the analysis of fatty acids in cow’s milk. The response factors (RF) for GC analysis using FID were calculated. For most acids, RFs were close to 1, with the exception of short-chain fatty acids (C4:0–C8:0). To facilitate the selection of the method for the determination of fatty acids in milk samples, the methods were assessed using the environmental assessment tools of the analytical procedure: the Analytical Eco-Scale, Green Analytical Procedure Index (GAPI), and Analytical Greenness for Sample Preparation (AGREEprep). The method based on direct milk methylation received the highest scores. Omitting the lipid separation step has an impact on reducing the quantity of used toxic chemicals and reagents, and produces a smaller amount of waste, a much higher throughput, and a reduced cost analysis.

## 1. Introduction

Milk is a nutrient-rich food source in the human diet which contains lipids (dairy fat), high-quality protein, vitamins, minerals, and other bioactive components [1]. The most valuable component of milk is fat because it directly affects the nutritional value of the product, and also has an effect on sensory properties such as flavor and aroma [2]. Majority of milk lipids are in the form of triacylglycerols (TGA), which consist of a molecule of glycerol bound to three fatty acids (FA). When triacylglycerol is digested, then FAs become available for the human organism [3]. The FA composition is one of the most important indicators of the nutritional quality and physicochemical properties of milk fat. In addition to being related to human health, milk FAs can act as a potential indicator for the energy balance, metabolism and health of lactating cows, and can be used to predict new characteristics such as methane emissions and energy balance [4,5]. The FA composition of milk fat is influenced by various factors such as animal genotypes, diet, lactation stage and the physiological state of the cows. As a result, analysis of the composition of FA is of great importance in lipid-related research and for the dairy industry [6,7].

Generally, FAs are quantified according to their methyl esters (FAMEs) by gas chromatography-flame ionization detector (GC-FID) or gas chromatography-mass spectrometry (GC-MS) following a sample preparation procedure, i.e., lipid extraction and transesterification. Methods used for methyl esterification include acid or base catalysis, as also acetyl chloride-methanol catalysis, BF3, or other agents. However, the acid- or alkaline-catalysis are most widely used for the determination of FA. The acid-catalyzed methylation can convert FA from all lipid classes present in a sample into their correspondent FAME, but the methylation process is slower than alkaline-catalyzed methylation and could modify the profile of conjugated linoleic acids (CLA). In the case of alkaline-catalyzed methylation, the main drawback this method is that only acyl moieties are converted to FAME. However, this does not cause important bias in the results because the proportion of lipids other than acyl moieties is low in milk fat. As mentioned above, the alkaline-catalyzed methylation is faster than acid-catalyzed methylation. Therefore, the alkaline-catalyzed methylation is often recommended for milk FA profiling [6,8].

There are several methods of preparing samples of cow’s milk for the determination of FA, e.g., the Folch method or Rose–Gottlieb method [9,10]. Methodological comparison studies for the determination of FAs are very influential, because milk samples require extreme care to ascertain the lipid fraction, given that factors such as co-extraction of non-fatty component lipids and undesirable oxidation may influence the quality and final quantification of the lipid fraction [11]. These methods are characterized by the consumption of toxic reagents, and are time-consuming and labor-intensive.

Therefore, it is important to evaluate the greenness of analytical procedures to assess, and, if possible, reduce their impact on the environment and workers. Several tools are used in green analytical chemistry to address the environmental performance of an analytical procedure, including Analytical Eco-Scale, National Environmental Method Index (NEMI), Green Analytical Procedure Index (GAPI), and Analytical Greenness for Sample Preparation (AGREEprep), in which objective criteria related to analytical performance, sustainability, environmental impact, and economic cost are evaluated through the definition of penalty points [12,13].

The main task of the presented study was to compare eight selected methods for the analysis of fat content in cow’s milk. Additionally, the methods themselves were assessed using the environmental assessment tools of the analytical procedure. The results of these tests may be useful for researchers, and persons performing routine milk analyses in deciding on the choice of the analytical procedure.

## 2. Results and Discussion

### 2.1. Optimization of GC-FID Conditions

**Figure 1:**
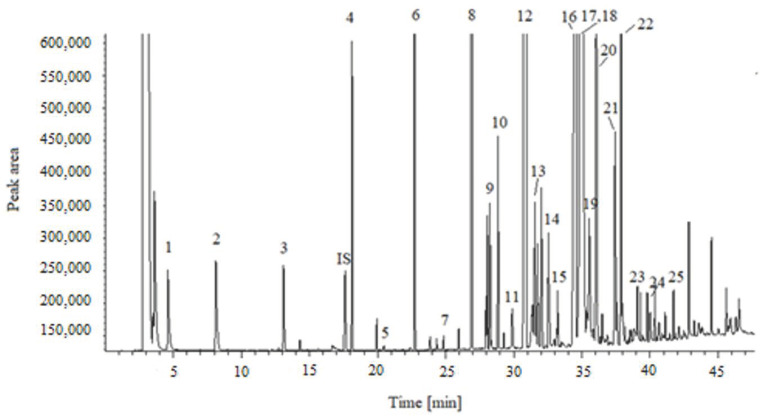
Typical chromatogram of milk lipid FAMEs. Peaks are: 1. butyric acid, 2. caproic acid, 3. caprylic acid, 4. capric acid, 5. undecanoic acid, 6. lauric acid, 7. tridecanoic acid, 8. myristic acid, 9. myristoleic acid, 10. pentadecanoic acid, 11. cis-100-pentadecanoic acid, 12. palmitic acid, 13. palmitoleic acid, 14. heptadecanoic acid, 15. cis-heptadecanoic acid, 16. stearic acid, 17. elaidic acid, 18. oleic acid, 19. linolelaidic acid, 20. linoleic acid, 21. arachidic acid, 22. linolenic acid, 23. cis-11-eicosenoic acid, 24. behenic acid, 25. arachidonic acid, IS. internal standard.

Preliminary investigations aimed for adjustment and selection of the chromatographic conditions for the GC-FID analysis of FAs in milk. The research carried out by the manufacturer of the standard (37 FAMEs standard) and the column [14] were used to identify FAs. Separation and identification of over 20 different FAs was achieved, ranging from short-chain (C4:0) to long-chain (C22:0), particularly including various branched-chain FAs, C:18:1 isomers, and CLAs. With the exception of compounds 17/18 (elaidic and oleic acids), all of the compounds were baseline separated. The unseparated peak from elaidic and oleic acids did not affect the purpose of this study. For the determination of the fatty acid profile in milk, fatty acids present in the sample above 0.01 g/100 g FA were taken into account. A typical chromatogram of FAMEs from cow milk is shown in Figure 1.

The used of FID for the quantification of FAMEs is advantageous in relation to other detector types, because it is stable and easy to operate, possesses a wide dynamic range, and its introduction and maintenance costs are lower than for other types of detectors. The FID response is proportional to the number of carbon atoms that are burned. Heteroatoms (e.g., oxygen) in molecules usually reduce the FID signal which can worsen accuracy in the quantification of fatty acids analysis. In these cases, in order to correct the responses of the detector, a response factor relative to each one of the analytes with respect to an internal standard is used [15,16].

In our research, experimental response factors (ERFs) for the quantification of individual FAs were determined by using standard FAMEs in the appropriate concentration. Table 1 gives the ERF and theoretical correction factors (TRFs) and the error factor (EF). The determined ERFs were compared with the TRFs. The ideal is to obtain results with an EF close to one, as in this way the results obtained will be highly accurate. Almost all of the ERFs were lower than the TRFs, which could have resulted from improper functioning of the GC system, purity of the standards, adsorption, decomposition, or discrimination of analytes during GC. In particular the difference is seen for C4:0 and C6:0 due to losses of volatility during the preparation of calibration solutions. With the exception of these two acids, the ERF results ranged from about 0.9–1.0. Therefore, we followed the recommendation of Bannon et al. [17] to use the theoretical factors in quantitative determinations of FAs, when there is a significant difference between REF and TRF.

The precision of the quantitative method was evaluated through the repeatability (intra-day) and reproducibility (inter-day) experiment. The intra-day part of the method was established from six complete analyses of each sample under the same conditions in a day, and the inter-day was established from three complete analyses of each sample repeated on three consecutive days. Both intra-day and inter-day precision were satisfactory. The intra-day RSD ranged between 0.6 and 8.8% and inter-day RSD ranged between 0.5 and 10.0% (Table 1).

### 2.2. Comparison of Preparation Methods for FAMEs Determination

In the literature [4,6,18,19,20] and international standards [21,22,23], there are many methods for the determination of FAs in a milk sample. Typically, FAMEs are quantified by GC following the multi-step sample preparation procedure. The standard procedure for determining the profile of FAs in milk fat usually consists of the isolation of fat (extraction, centrifugation, evaporation), and the transesterification and extraction of FA.

Liu, Ezernieks, Rochfort, & Cocks [24] compared the transesterification methods of FA. This research shows the advantages and disadvantages of acid- and alkaline-catalyzed transesterification. The results presented by the researchers show no significant differences (*p* > 0.05) between the different methods of methylation for the majority of FAs. However, due to the shorter time in alkaline-catalyzed methylation, this type of transesterification was chosen in our research.

In our work, we used eight methods that differ mainly in the way of the fat isolation from milk sample (Table 2). In order to obtain fat, extraction with organic solvents (methods A, G and G’), centrifugation (C, D), and centrifugation combined with evaporation (B) were used. Additionally, we used the method of directly methylation in milk (E and F), which was presented by Liu et al. [6]. The steps of sample preparation procedures used in our work was presented in Figure S1 (Supplementary Material).

As noted by Liu et al. [4], the use of crude fat isolated by centrifugation gives similar results in the relative proportion of each single FA compared to classical protocols requiring lipid extraction with organic solvents, which was also confirmed in our work (methods A, B, C and D). These methods also did not differ significantly (*p* < 0.05) from methods A, B, C and D. For methods B, C and D, which used centrifugation of the milk sample to extract the milk fat, it was found that the milk fat contained a significant amount of water. In order to check the influence of the presence of water in the fat on the obtained results, the analysis of fresh fat (method B) and dried fat (method C) was performed. It was found that for most of the main FAMEs monitored there was no significant difference (*p* > 0.05) between fresh and dry fat. This suggests that although the centrifuged milk fat contains a significant amount of water, it does not affect the methylation reaction. Nevertheless, it should be emphasized that due to the presence of water and other components in the centrifuged fat layer, direct methylation of a weighed sample of crude fat does not allow for a reliable calculation of the absolute content of individual FAs in milk (mg FA/100 g fat). In order to obtain the content of the FA in the milk fat, the content of water and other interfering substances would have to be precisely determined and included in the calculations.

The results of FA content in milk obtained by direct methylation methods (E and F) do not differ significantly (*p* > 0.05) from methods in which fat isolation (A–D) is used. Compared to method F, method E additionally used evaporation of the milk sample before the methylation step, which resulted in a slight loss of volatile FA. However, they were not affected significantly (*p* > 0.05) in the results. It should be pointed out that direct methylation of liquid milk (in E and F methods) did not make any additional interfering peaks as compared to the solvent-extracted ‘clear’ lipids. It is important because extra peaks can often co-elute, interfere with, and, thus, compromise the integration accuracy of peaks. Comparing the presented methods on the basis of the sample preparation steps (Figure S1, Supplementary Material), it can be concluded that the E and F methods are the safest, least time-consuming and cheapest methods. The skipping of the lipid extraction step and the possibility of direct transesterification of FAs is very advantageous compared to other methods.

Compared to the Folch (A) and modified PN-ISO 15885 (D) methods, which contained only lipids, the reaction matrices were much more complex when liquid milk was used directly for FAME preparation. However, despite the different behavior of the incubation samples (i.e., for method A—the reaction mixture was clear, in method B—milk solids clung to the wall of the vial, in method C—the reaction mixture was a cloudy suspension, for method E and F—residual milk solids could be seen in the reaction mixture), in all cases, upon adding hexane, phase separation was always achieved, and a transparent hexane extract obtained.

The overall GC-FID profile of FAMEs is similar for all major FA across the six above-mentioned compared methods. However, the results obtained for the G–G‘ methods are significantly different (*p* < 0.05) from the other methods (A–F). This may be due to the multiple steps of the procedure, which may result in the loss of some FAs. Therefore, it can be concluded that incorrect results can be obtained when using method G for the determination of the FA profile.

### 2.3. Assessment of the Method Greenness

In order to facilitate the selection of the most advantageous method, it was decided to evaluate them in terms of their greenness. The objective is to evaluate the green impact of used methods sample preparation on operators and the environment. This assessment includes the characteristics and amount of solvents and reagents used, amounts of waste produced, energy consumption, and the duration of the study.

For the assessment we used three various matrices to estimate the greenness of our compared methods such as the Analytical Eco-scale, Green Analytical Procedure Index (GAPI), and Analytical Greenness Metric for Sample Preparation (AGREEprep). The Analytical Eco-Scale was introduced in 2012 by Gałuszka et al. [25]. In this method we calculate penalty points (PPs), which are assigned for high amounts, and high hazards connected with utilization of chemicals, high energy consumption, occupational hazards, and generation of wastes. The final result of an analytical Eco-Scale assessment is a number differing from 100 (‘ideal green analysis’) by a number of PPs. If the final score is above 75 points, it is considered ‘excellent green analysis’, but if it is between 50 and 75 points, it is considered ‘acceptable green analysis’. The method with a final result below 50 points, is deemed ‘inadequate green analytical procedure’ [26,27]. The PPs for all the methods (A–G’) are presented in Table 3, and the details of scoring are demonstrated in Table S1 (Supplementary Material).

The GAPI was introduced in 2018 by Płotka-Wasylka [28]. This is a new tool to assess the green character of the entire analytical procedure. GAPI’s visual presentation allows for easy comparison of various methods and selecting from them the greenest. GAPI includes five pentagrams with 15 investigated parameters that describe the environmental impact of every step of the analytical methodology, such as sample collection and preparation, health and safety impact of reagents and compounds used, waste treatment, and energy consumption by instrumentation. The description of the pentagram is presented in Figure S2 (Supplementary Material). GAPI uses a three-level color scale: green, yellow, and red to represent low, medium, and high ecological impact for each step. The greenest method is that possessing the highest number of green zones, and least number of red zones. The green assessment profiles for the methods using the GAPI tool are presented in Table 3. Detailed descriptions of GAPI parameters for the methods are shown in Table S2 (Supplementary Material).

Recently, in 2022, AGREE creators introduced a modification to it called AGREEprep [29]. The proposed metric tool gives prominence to sample preparation only. The AGREEprep was based on 10 categories (description in the Supplementary Material) of impact that were recalculated to 0–1 scale sub-scores. Assessment was also based on the possibility to differentiate between criteria importance by assigning them weights. The assessment produces a pictogram summarizing the overall greenness of the method. The criteria of assessment evaluated, among others, the choice and use of solvents, materials and reagents, waste generation, energy consumption, sample size, and throughput [13]. The pictograms made in the AGREEprep assessment are presented in Table 3. Detailed reports of these assessments are available in the Supplementary Material.

Taking into account the complexity of the matrix, which is the milk, and the number of analytes determined, as well as the necessity to perform derivatization, one cannot expect high grades of the greenness of these methods. According to Eco-Scale, the A–F methods achieved 70–73 points, which qualifies them as ‘acceptable green analysis’. In contrast, the G and G’ methods obtained less than 50 PPs and, therefore, belong to the group of “inadequate green analysis”.

Methods A, B, and C were assessed identically by GAPI. On the other hand, slight differences in the evaluation of these methods are visible when using the Analytical Eco-Scale and AGREEprep methods. The E and F methods were rated the highest by all the evaluation methods used. In the case of these two methods, the highest convergence of results was obtained, which for Analytical Eco-Scale and GAPI are identical. Slight differences in their assessment can be noticed when using the AGREEprep, where the result for the E method was 0.33, and for the F method the result was 0.34. This slight difference is due to the fact that method F was created by simplifying method E by omitting the step of evaporating the water and derivatizing the FA directly in the milk. From this it can be concluded that AGREEprep is the most accurate way to assess the ‘greenness’ of analytical methods. It is probably related to the greater influence of even a small amount of used reagents and other parameters on the calculated final result. However, in the case of Analytical Eco-Scale and GAPI, e.g., in the category of the amount of reagents used, the same score is obtained in the range of 10–100 mL. The E and F methods proposed by Liu et al. [6] are eco-friendly, mainly due to the use of a small amount of sample (200 µL) and small amounts of toxic reagents. Additionally, and of great importance, they are the least labor-consuming and time-consuming.

The methods G and G’ [21] in the evaluation of greenness obtained very bad results. They obtained a large amount of PPs—76, which resulted in the final result being 24. The modification performed by us (method G’) consisting in reducing the amount of the sample used for the test and the amount of appropriate reagents by 90%, and lowering the PPs to 55, gave a result of 45. However, both methods (G, G’) have been classified by the Analytical Eco-Scale as ‘inadequate green analytical procedure’. Their assessment by GAPI, and AGREEprep was also very disadvantageous. The 15-field GAPI pictograms contain 11 and 9 red fields for G and G’ methods, respectively. Likewise, almost all AGREEprep pictograms are red, and their ratings are very low at 0.04 (G) and 0.06 (G’). Such results are related to the use of large amounts of toxic solvents and waste, a large amount of sample for testing, and the multistage and time-consuming of the procedure. It was not approved by any of the assessment methods used. This assessment allows for the conclusion that the use of this method should be avoided.

Summing up the evaluation of the greenness of the tested methods, it can be stated that it should be taken into account before making a decision on the selection of the sample preparation method for the routine analysis of FA content in milk samples. Generally, all the discussed tools can assess the ‘greenness’ of analytical protocols and have their inherent merits and drawbacks, and, hence, the ideal solution is to implement two of them at least to extract the maximum possible information about analytical procedures.

## 3. Materials and Methods

### 3.1. Milk Sample

Raw cow milk was purchased from a local farm (Pomerania, Poland). This sample was an aliquot from an afternoon milking of one cow; its total fat concentration was 3.9% as determined by infrared spectroscopy. Sample was frozen immediately after collection and stored at −18 °C until use.

### 3.2. Chemicals and Reagents

Solvents and chemicals used for lipid extraction and FAME preparation were of analytical grade. Chloroform, methanol, n-hexane, ethanol, n-pentane, ammonia, diethyl ether, anhydrous sodium sulphate, disodium hydrogen citrate sesquihydrate, potassium hydroxide and sodium hydroxide, and the hexadecane (used as internal standards) were from Sigma Aldrich (Darmstadt, Germany). The standard mix of 37 FAMEs and standard mix of C4:0–C24:0 even-carbon saturated FAMEs were purchased from Supelco (Bellefonte, PA, USA).

### 3.3. Lipid Extraction and FAME Preparation

The study compares eight methods of isolating fat from raw milk. All methods as prescribed in the literature and international standards.

Method A (Folch method) [4,9]: total lipids of raw milk (0.5 mL) were extracted twice by chloroform/methanol (2:1, *v*/*v*), and the organic phase was transferred to vial and evaporated to dryness under a stream of N_2_. Next, 2.4 mL of derivatization reagent (0.2 M KOH/MeOH) was added, and sample was incubated at 50°C for 20 min. After cooling, 1 mL of water was added, and FAMEs formed were extracted into 1 mL of n-hexane and subjected to GC-FID analysis. The total time of sample preparation was 60 min.

Method B [4]: raw milk (14 mL) was centrifuged for 20 min and 25–30 mg of crude fat was weighted into a vial, and dried under a stream of N_2_ for 60 min before methylation. The methylation reaction and the rest of the method were performed as described in the Method A. The total time of sample preparation was 120 min.

Method C [4]: raw milk (14 mL) was centrifuged for 20 min and 25–30 mg of crude fat was weighted into a vial, and directly subjected to methylation. The methylation reaction and the rest of the method were performed as described in the Method A. The total time of sample preparation was 60 min.

Method D [30]: raw milk (15 mL) was centrifuged for 20 min and 50 mg of crude fat. Then, 5 mL of 5% (m/v) CH_3_ONa solution was added. Next, the tube was shaken well for 10 s. A total of 180 s after the start time, tube was opened, and added 2 mL of n-hexane. Then, 210 s after start time, 10 mL of disodium hydrogen citrate and sodium chloride aqueous solution was added and shaken gently for 30 s. Then, the sample was centrifuged for 20 min. Supernatant was subjected to GC-FID analysis. The total time of sample preparation was 60 min.

Method E [6]: fresh milk (200 µL) was measured into a vial and then dried in a heating block (40 °C) under a stream of N_2_ for approximately 15 min. The 2.5 mL of derivatization reagent (0.2M KOH/MeOH) was added, and a sample was incubated at 50 °C for 30 min with occasional shaking. After cooling to room temperature, 1 mL of HCL (1 M) was added to each vial and FAMEs formed were extracted into 1 mL of n-hexane and analyzed directly by GC-FID. The total time of sample preparation was 60 min.

Method F [6]: fresh milk (200 µL) was subjected directly to methylation in a 5 mL glass vial without any pre-treatment. The methylation reaction and the rest of the method were performed as described in the Method E. The total time of FAME preparation this method was 40 min.

Method G [31]: 100 mL of the milk sample was mixed with 80 mL of EtOH and 20 mL of NH_3_ solution in a funnel. Then, 100 mL of diethyl ether was added, and the funnel was shaken vigorously for 1 min. The solution was stood to achieve phase separation. Next, 100 mL of n-pentane was added to the solution in the funnel and was mixed carefully. Then, after phase separation, the aqueous layer was discarded. Next, a 100 mL of sodium sulfate solution was added to the organic phase. After phase separation, the aqueous layer was discarded. The procedure with sodium sulfate solution was repeated twice. Then, 10 g of anhydrous sodium sulfate was added to the organic phase, and the content was mixed carefully. The flask was stand for 10 min and its contents was filtered. Using the rotary evaporator and stream of N_2_, content of the flask was evaporated. Then, 100 mg of the sample was weighted and dissolved in a 5 mL of n-hexane and mixed. A total of 0.2 mL of the transesterification reagent (2M KOH/MeOH) was added, and mixed with the vortex mixer for 1 min. After, the additional reaction time of 5 min, 0.5 g of solid sodium sulfate was added and mixed again. The test tube was centrifuged for 3 min at room temperature. Supernatant was subjected to the GC-FID analysis. The total time of sample preparation was 180 min.

Method G’ [31]: The preparation method was performed as described in Method G. However, this method used 10 times smaller the amount of sample and reagents for preparation method. The total time of sample preparation was 180 min.

In order to illustrate the course of proceedings in the abovementioned methods, a flow chart was created (Figure S1, Supplementary Materials).

### 3.4. GC Analysis

Chromatographic analyses were performed using an Agilent 7890B (Agilent, Santa Clara, CA, USA), equipped with a flame ionization detector (FID), split/splitless injector, and multipurpose autosampler. The GC was fitted with a SP-2380 column, 30 m × 0.25 mm × 0.2 µm (Supelco, Bellefonte, PA, USA), with a constant flow of 1.0 mL/min helium as carrier gas.

The injector port was held at 230 °C and used in the split mode using a split ratio of 10:1, and injection volumes were 1 µL. The detector temperature was 250 °C. The oven temperature program was 50 °C, where it was held for 2 min, then increasing it at 4 °C/min to 220 °C and held for 15 min.

### 3.5. Statistical Analysis

Peak areas were obtained by manual integral with Agilent ChemStation F.01.00.1903 (Agilent Technologies, Santa Clara, CA, USA). The experiments were carried out at least three times, and the results were expressed as the mean ± standard deviation. The data were subjected to analysis of variance (ANOVA) and Tukey’s test. Results were considered statistically significant at *p* < 0.05.

### 3.6. Calculation of Fatty Acid Contents

FAMEs were identified by comparison of retention times with reference standards (37 FAMEs and C4:0-C24:0 even carbon saturated FAMEs, SUPELCO) analyzed under the same conditions.

Peak areas were corrected by correction factor (F*_i_*) described in ISO 12966-4:2015 [30].

The correction factor, F*_i_*, is then:

(1) $$F_{i}=\frac{m_{i}\times \sum A}{A_{i}\times \sum m}$$

where:

m*_i_*—is the mas of FAME, *i*, in the reference mixture,
∑A—is the sum of all areas of all FAMEs of the reference mixture,
A*_i_*—is the area of FAME, *i*, in the reference mixture,
∑m—is the total of the masses of the various components, as FAMEs of the reference mixture.

For the sample, the mass fraction, w*_i_*, in grams per 100 g of each FAME, *i*, is as given by formula:

(2) $$w_{i}=\frac{F_{i}\times A_{i}}{\sum \left(F_{i}\times A_{i}\right)}$$

The calculated value corresponds to the percentage of mass of the individual FA calculated as triacylglycerol per 100 g fat:

(3) $$\mathrm{FA}\left(\%\right)=\frac{F_{i}\times A_{i}}{\sum \left(F_{i}\times A_{i}\right)}\times 100$$

## 4. Conclusions

The procedure of preparing a milk sample for the determination of the FA profile is usually multi-step, which makes it labor-intensive and time-consuming. Most often it requires the separation of fat by extraction or centrifugation, the use of transesterification reactions, and extraction of the separated acids. For this purpose, the use of solvents and toxic reagents, the consumption of energy, and the production of hazardous waste is necessary. There are many procedures available in the literature that differ mainly in the derivatizing agent, such as alkaline or acidic agents, BF_3_ and others. The paper presents the results of the comparison of studies on the FA profile in a sample of cow’s milk determined with the use of eight methods available in the literature that use alkaline methylation. In order to facilitate the selection of the method used for routine analyses of the FA profile in milk, an assessment of the environmental impact of these methods was made. For this purpose, three methods of greening assessment were used: Analytical Eco-scale, Green Analytical Procedure Index (GAPI), and Analytical Greenness Metric for Sample Preparation (AGREEprep). As expected, none of the methods belong to the green procedures. The method in which methylation of FA is carried out directly in milk was scored the highest. The environmental assessment tools of the analytical procedure should be effectively compared and incorporated as a standard in the development and validation of a new environmentally benign analytical method.

## Figures and Tables

**Table 1:** Experimental and theoretical correction factors, error factor, intra-day, and inter-day precision for the fatty acid in milk sample.

Fatty Acid		ERF ^1^	TRF ^2^	EF ^3^	Intra-Day RSD (%)	Inter-Day RSD (%)
butyric acid	C4:0	2.3260	1.5742	1.4776	7.5	8.0
caproic acid	C6:0	1.5308	1.3378	1.1443	6.6	2.4
caprylic acid	C8:0	1.1036	1.2195	0.9050	4.1	1.8
capric acid	C10:0	0.9640	1.2702	0.7589	2.5	1.7
undecanoic acid	C11:0	0.9501	1.1486	0.8272	2.3	2.9
lauric acid	C12:0	0.9433	1.1013	0.8566	0.6	1.3
oleic acid	C13:0	0.9549	1.0831	0.8817	1.2	0.9
myristic acid	C14:0	0.9678	1.0675	0.9066	1.6	0.8
myristoleic acid	C14:1	0.9835	1.0587	0.9290	1.6	1.0
pentadecylic acid	C15:0	0.9514	1.0540	0.9026	1.1	0.7
ginkgolic acid	C15:1	0.9439	1.0457	0.9027	1.0	1.7
palmitic acid	C16:0	0.9418	1.0422	0.9037	2.0	0.6
palmitoleic acid	C16:1	0.9909	1.0345	0.9579	1.7	0.5
heptadecanoic acid	C17:0	0.9418	1.0318	0.9127	1.9	1.1
10-heptadecenoic acid	C17:1	0.9019	1.0244	0.8804	1.7	4.5
stearic acid	C18:0	0.9183	1.0225	0.8981	3.5	1.6
elaidic acid + oleic acid	C18:1n9t + C18:1n9c	0.9000	1.0155	0.8863	1.8	1.1
linolealidic acid	C18:2n6c	0.9510	1.0087	0.9428	8.8	1.4
linoleic acid	C18:2n6t	0.9112	1.0087	0.9033	1.7	6.0
arachidic acid	C20:0	0.9813	1.0067	0.9748	1.4	6.6
alpha-linolenic acid	C18:3n3	0.9060	1.0017	0.9044	8.9	1.6
11-eicosenoic acid	C20:1n9	0.9359	1.0005	0.9354	7.3	10.0
behenic acid	C22:0	0.9326	0.9939	0.9384	2.9	2.7
arachidonic acid	C20:4n6	1.0020	0.9819	1.0205	2.4	5.8

^1^ ERF = Experimental response factor, ^2^ TRF = Theoretical response factor, ^3^ EF = Error factor (ERF/TRF).

**Table 2:** Fatty acid compositions of cow milk. Data are expressed as total mean (g/100 g FA) ± standard deviation of samples.

Nr of FA	FA	Method A	Method B	Method C	Method D	Method E	Method F	Method G	Method G’
1	C4:0	1.19 ± 0.05 ^c^	1.46 ± 0.01 ^a^	1.41 ± 0.01 ^a^	1.83 ± 0.05 ^d^	1.43 ± 0.08 ^a^	1.57 ± 0.06 ^a^	3.97 ± 0.09 ^b^	3.87 ± 0.11 ^b^
2	C6:0	1.69 ± 0.09 ^a^	1.79 ± 0.14 ^a^	1.68 ± 0.00 ^a^	2.24 ± 0.04 ^a^	1.83 ± 0.12 ^a^	1.90 ± 0.02 ^a^	2.05 ± 0.10 ^a^	2.15 ± 0.80 ^a^
3	C8:0	1.12 ± 0.05 ^a^	1.07 ± 0.08 ^ac^	1.02 ± 0.04 ^a^	1.27 ± 0.03 ^a^	1.19 ± 0.07 ^a^	1.11 ± 0.02 ^a^	0.97 ± 0.07 ^ab^	0.95 ± 0.04 ^bc^
4	C10:0	2.66 ± 0.05 ^a^	2.52 ± 0.09 ^a^	2.42 ± 0.06 ^ac^	2.72 ± 0.02 ^a^	2.79 ± 0.05 ^a^	2.84 ± 0.09 ^a^	2.06 ± 0.15 ^b^	2.16 ± 0.19 ^bc^
5	C11:0	0.06 ± 0.00 ^a^	0.06 ± 0.00 ^a^	0.06 ± 0.00 ^a^	0.07 ± 0.00 ^b^	0.08 ± 0.00 ^bc^	0.08 ± 0.01 ^c^	0.05 ± 0.00 ^d^	0.03 ± 0.00 ^e^
6	C12:0	3.31 ± 0.02 ^a^	3.13 ± 0.02 ^a^	3.06 ± 0.06 ^a^	3.19 ± 0.01 ^a^	3.38 ± 0.15 ^a^	3.35 ± 0.15 ^a^	2.53 ± 0.16 ^b^	2.46 ± 0.11 ^b^
7	C13:0	0.13 ± 0.00 ^a^	0.12 ± 0.00 ^a^	0.12 ± 0.00 ^a^	0.12 ± 0.00 ^a^	0.13 ± 0.01 ^a^	0.14 ± 0.01 ^a^	0.10 ± 0.01 ^b^	0.09 ± 0.01 ^b^
8	C14:0	11.99 ± 0.04 ^a^	11.57 ± 0.14 ^a^	11.46 ± 0.18 ^a^	11.26 ± 0.06 ^a^	11.93 ± 0.40 ^a^	11.50 ± 0.37 ^a^	9.77 ± 0.38 ^b^	9.54 ± 0.39 ^b^
9	C14:1	1.61 ± 0.01 ^a^	1.54 ± 0.02 ^a^	1.52 ± 0.03 ^a^	1.57 ± 0.01 ^a^	1.58 ± 0.05 ^a^	1.59 ± 0.04 ^a^	1.06 ± 0.06 ^b^	1.09 ± 0.07 ^b^
10	C15:0	1.30 ± 0.00 ^a^	1.27 ± 0.02 ^a^	1.27 ± 0.02 ^a^	1.25 ± 0.01 ^a^	1.30 ± 0.04 ^a^	1.25 ± 0.02 ^a^	1.13 ± 0.03 ^b^	1.11 ± 0.02 ^b^
11	C15:1	0.26 ± 0.00 ^a^	0.26 ± 0.01 ^a^	0.26 ± 0.00 ^a^	0.25 ± 0.00 ^a^	0.26 ± 0.00 ^a^	0.25 ± 0.00 ^a^	0.23 ± 0.00 ^b^	0.21 ± 0.00 ^b^
12	C16:0	31.34 ± 0.10 ^a^	32.11 ± 0.79 ^a^	31.87 ± 0.83 ^a^	30.49 ± 0.15 ^a^	31.58 ± 0.48 ^a^	31.71 ± 0.30 ^a^	29.73 ± 0.18 ^b^	29.52 ± 0.27 ^b^
13	C16:1	2.26 ± 0.01 ^a^	2.00. ± 0.04 ^b^	2.01 ± 0.04 ^b^	2.01 ± 0.03 ^b^	2.08 ± 0.16 ^ab^	1.95 ± 0.04 ^b^	1.67 ± 0.03 ^c^	1.62 ± 0.03 ^c^
14	C17:0	0.69 ± 0.01 ^a^	0.71 ± 0.02 ^a^	0.72 ± 0.01 ^a^	0.63 ± 0.00 ^ac^	0.70 ± 0.00 ^a^	0.71 ± 0.03 ^a^	0.61 ± 0.01 ^bc^	0.58 ± 0.01 ^b^
15	C17:1	0.29 ± 0.00 ^ac^	0.27 ± 0.01 ^a^	0.28 ± 0.02 ^a^	0.29 ± 0.00 ^a^	0.28 ± 0.00 ^a^	0.27 ± 0.01 ^a^	0.33 ± 0.00 ^b^	0.31 ± 0.00 ^bc^
16	C18:0	10.53 ± 0.09 ^a^	11.3 ± 0.39 ^a^	11.27 ± 0.38 ^a^	10.23 ± 0.00 ^a^	10.17 ± 0.10 ^a^	10.50 ± 0.15 ^a^	13.61 ± 0.67 ^b^	13.29 ± 0.35 ^b^
17 + 18	C18:1n9t + C18:1n9c	25.76 ± 0.31 ^a^	25.7 ± 0.63 ^a^	26.35 ± 0.42 ^a^	26.31 ± 0.09 ^a^	25.35 ± 0.44 ^a^	25.69 ± 0.72 ^a^	24.69 ± 0.85 ^a^	24.89 ± 0.47 ^a^
19	C18:2n6c	2.25 ± 0.05 ^ac^	2.22 ± 0.04 ^ac^	2.24 ± 0.01 ^ac^	2.27 ± 0.03 ^ac^	2.19 ± 0.02 ^a^	2.21 ± 0.01 ^ab^	2.52 ± 0.06 ^d^	2.31 ± 0.05 ^bc^
20	C18:2n6t	0.49 ± 0.01 ^a^	0.49 ± 0.01 ^a^	0.51 ± 0.01 ^a^	0.49 ± 0.00 ^a^	0.48 ± 0.01 ^a^	0.47 ± 0.03 ^a^	1.05 ± 0.08 ^b^	1.03 ± 0.04 ^b^
21	C20:0	0.18 ± 0.00 ^a^	0.19 ± 0.01 ^a^	0.19 ± 0.01 ^ab^	0.16 ± 0.00 ^a^	0.18 ± 0.00 ^ab^	0.21 ± 0.00 ^bd^	0.26 ± 0.01 ^e^	0.23 ± 0.01 ^d^
22	C18:3n3	0.46 ± 0.01 ^a^	0.44 ± 0.01 ^a^	0.45 ± 0.00 ^a^	0.47 ± 0.00 ^a^	0.44 ± 0.01 ^a^	0.45 ± 0.01 ^a^	0.45 ± 0.01 ^a^	0.39 ± 0.01 ^b^
23	C20:1n9	0.53 ± 0.01 ^a^	0.54 ± 0.01 ^a^	0.54 ± 0.01 ^a^	0.55 ± 0.01 ^a^	0.52 ± 0.01 ^a^	0.54 ± 0.01 ^a^	0.76 ± 0.05 ^b^	0.72 ± 0.06 ^b^
24	C22:0	0.13 ± 0.00 ^a^	0.13 ± 0.00 ^a^	0.13 ± 0.00 ^a^	0.13 ± 0.00 ^a^	0.13 ± 0.00 ^a^	0.13 ± 0.00 ^a^	0.20 ± 0.02 ^b^	0.19 ± 0.02 ^b^
25	C20:4n6	0.18 ± 0.00 ^a^	0.18 ± 0.00 ^a^	0.18 ± 0.00 ^a^	0.19 ± 0.01 ^acd^	0.17 ± 0.00 ^a^	0.18 ± 0.00 ^ac^	0.21 ± 0.01 ^b^	0.20 ± 0.01 ^bd^
Sums									
ƩSFA ^1^		65.92 ^a^	66.35 ^a^	65.66 ^a^	65.60 ^a^	66.80 ^a^	66.90 ^a^	67.04 ^a^	67.11 ^a^
ƩUFA ^2^		34.08 ^a^	33.65 ^a^	34.34 ^a^	34.40 ^a^	33.20 ^a^	33.10 ^a^	32.96 ^a^	32.89 ^a^
ƩMUFA ^3^		30.70 ^a^	30.31 ^a^	30.97 ^a^	30.98 ^a^	30.08 ^ac^	30.29 ^ac^	28.73 ^bc^	28.89 ^bc^
ƩPUFA ^4^		3.38 ^a^	3.33 ^a^	3.38 ^a^	3.42 ^a^	3.12 ^a^	3.13 ^a^	4.23 ^b^	3.99 ^b^

^1^ ƩSFA = sum of saturated fatty acids; ^2^ ƩUFA = sum of unsaturated fatty acids; ^3^ ƩMUFA = sum of monounsaturated fatty acids; ^4^ ∑ PUFAs = sum of polyunsaturated fatty acids. Values are given as the means ± SD (*n* = 3). Different letters (^a–e^) in the same column indicate significant differences (*p* < 0.05).

**Table 3:** The greenness profile of the employed (A–G’) methods for analysis FAMEs in milk sample using Eco-Scale, GAPI, and AGREEprep metrics.

Method	Analytical Eco-Scale Score	GAPI Pictogram	AGREEprep Pictogram
A	70 acceptable green analysis	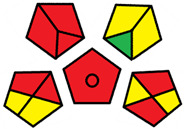 (1 green, 6 yellow, 8 red)	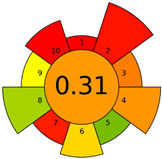
B	72 acceptable green analysis	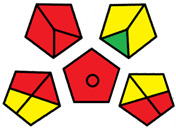 (1 green, 6 yellow, 8 red)	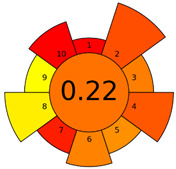
C	72 acceptable green analysis	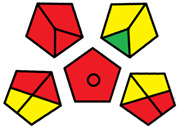 (1 green, 6 yellow, 8 red)	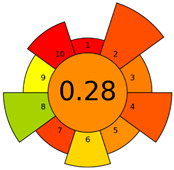
D	73 acceptable green analysis	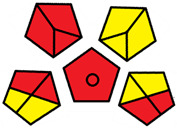 (0 green, 7 yellow, 8 red)	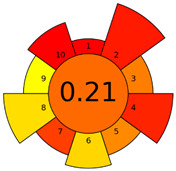
E	71 acceptable green analysis	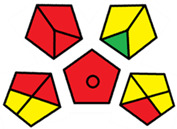 (1 green, 7 yellow, 7 red)	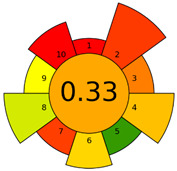
F	71 acceptable green analysis	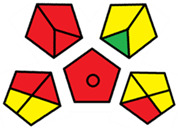 (1 green, 7 yellow, 7 red)	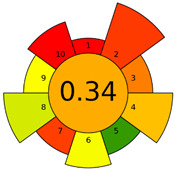
G	24 inadequate green analysis	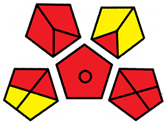 (0 green, 4 yellow, 11 red)	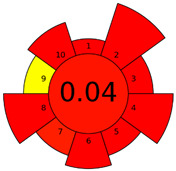
G’	45 inadequate green analysis	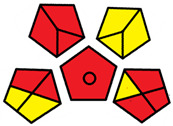 (0 green, 6 yellow, 9 red)	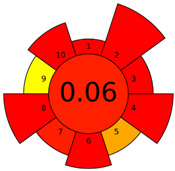

## Data Availability

Not applicable.

## Supplementary Materials

The following supporting information can be downloaded at: https://www.mdpi.com/article/10.3390/molecules27238242/s1, Figure S1: Scheme of the compared methods for the determination fatty acids in milk sample.; Figure S2: Green Analytical Procedure Index pictogram with description.; Table S1: Calculated PPs (Eco-Scale) for evaluated analytical procedures for FAMEs determination in milk samples.; Table S2: Green Analytical Procedure Index (GAPI) parameters for analytical procedures (A–D) for determination of FAME in milk samples.

## Sample Availability

Samples of the compounds are not available from the authors.
