# Supplementary material for: Comparison of the Effectiveness and Environmental Impact of Selected Methods for the Determination of Fatty Acids in Milk Samples
Source: Molecules. 2022 Nov 25;27(23):8242. doi: 10.3390/molecules27238242 (PMC9740020; doi:10.3390/molecules27238242)
Supplement: Supplementary file 1 [file molecules-27-08242-s001.zip › molecules-2014163-supplementary.pdf]

Supplementary Materials

Comparison of effectiveness and environmental impact of the selected methods for determination fatty acids in milk samples

Izabela Narloch and Grażyna Wejnerowska

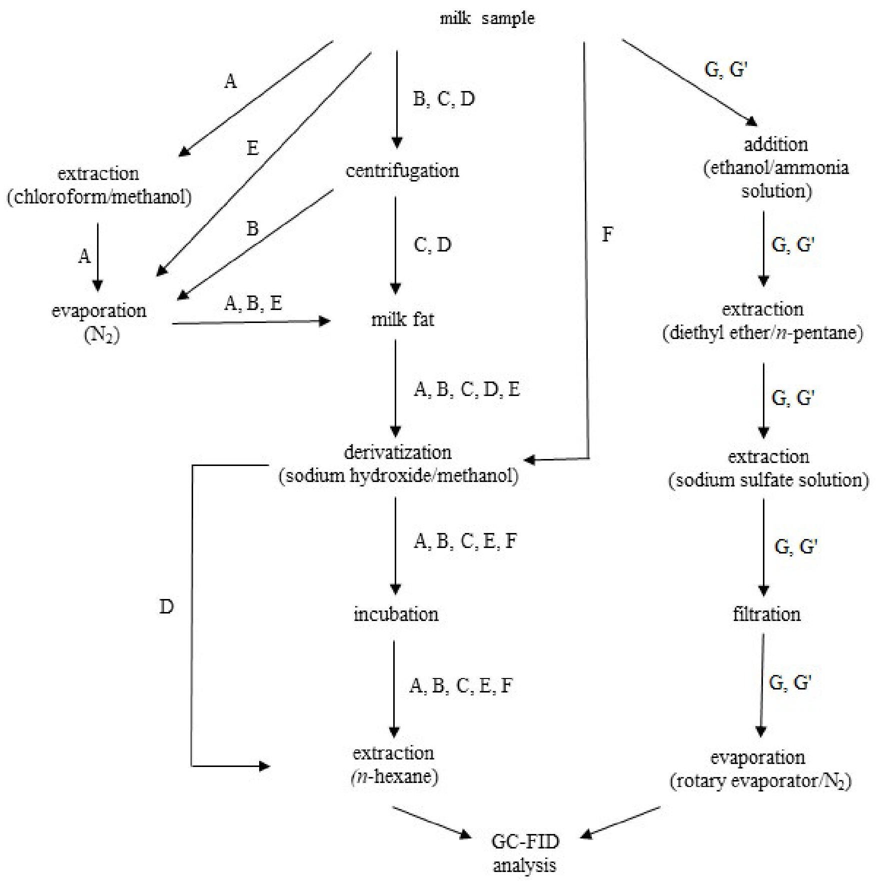

Figure S1. Scheme of the compared methods for the determination fatty acids in milk sample.

Table S1. Calculated PPs (Eco-Scale) for evaluated analytical procedures for FAMEs determination in milk samples (Procedures A - D).

**Procedure A**		**Procedure B**		**Procedure C**		**Procedure D**	
**Reagents**	**PPs**	**Reagents**	**PPs**	**Reagents**	**PPs**	**Reagents**	**PPs**
Methanol: 3.5 mL	6	Methanol: 2.1 mL	6	Methanol: 2.1 mL	6	Methanol: 4.75 mL	6
Chloroform: 3 mL	2	KOH: 0.3 g	2	KOH: 0.3 g	2	NaOH: 0.25 g	2
KOH: 0.3 g	2	n-hexane: 1 mL	8	n-hexane: 1 mL	8	n-hexane: 2 mL	8
n-hexane: 1 mL	8					Sodium hydrogencitrate/sodium chloride (3:2; *w:w*): 10 mL	0
	∑18		∑16		∑16		∑16
**Instrument**	**PPs**	**Instrument**	**PPs**	**Instrument**	**PPs**	**Instrument**	**PPs**
Transport	1	Transport	1	Transport	1	Transport	1
Sample storage (frozen)	0	Centrifugation (20 min; 5000 rpm)	1	Centrifugation (20 min; 5000 rpm)	1	Centrifugation (40 min; 5000 rpm)	1
Evaporation (N_2)	0	Evaporation (N_2, 60 min)	0	Incubation (50°C, 20 min)	1	GC-FID	1
Incubation (50°C, 20 min)	1	Incubation (50°C, 20 min)	1	GC-FID	1	Occupational hazard	0
Occupational hazard	3	Occupational hazard	0	Occupational hazard	0	Waste (>10 mL, no treatment)	8
GC-FID	1	GC-FID	1	Waste (>10 mL, no treatment)	8		
Waste (1-10 mL, no treatment)	6	Waste (>10 mL, no treatment)	8				
	∑12		∑12		∑12		∑11
**Total PPs**	**30**	**Total PPs**	**28**	**Total PPs**	**28**	**Total PPs**	**27**
**Score – acceptable green analysis**	**70**	**Score – acceptable green analysis**	**72**	**Score – acceptable green analysis**	**72**	**Score – acceptable green analysis**	**73**

Table S1. ***(cont.)*** Calculated PPs (Eco-scale) for evaluated analytical procedures for FAMEs determination in milk samples (Procedures E – G′).

**Procedure E**		**Procedure F**		**Procedure G**		**Procedure G′**	
**Reagents**	**PPs**	**Reagents**	**PPs**	**Reagents**	**PPs**		
Methanol: 2.1 mL	6	Methanol: 2.1 mL	6	Ethanol: 80 mL	3	Ethanol: 8 mL	2
KOH: 0.3 g	2	KOH: 0.3 g	2	diethyl ether: 100 mL	12	diethyl ether: 10 mL	8
HCl: 1 mL	4	HCl: 1 mL	4	n-pentane: 100 mL	24	n-pentane: 10mL	16
n-hexane: 1 mL	8	n-hexane: 1 mL	8	NH_3 (aq.): 20 mL	12	NH_3 (aq.): 2 mL	6
				10% Na_2SO_4(aq.): 200 mL	0	10% Na_2SO_4(aq.): 20 mL	0
				n-hexane: 50 mL	4	n-hexane: 5 mL	2
				KOH: 0.16 g	2	KOH: 0.016 g	2
				Methanol: 2 mL	6	Methanol: 0.2 mL	6
	∑20		∑20		∑63		∑42
**Instrument**	**PPs**	**Instrument**	**PPs**	**Instrument**	**PPs**	**Instrument**	**PPs**
Transport	1	Transport	1	Transport	1	Transport	1
Incubation (50°C, 30 min)	1	Incubation (50°C, 30 min)	1	Rotary evaporator	0	Rotary evaporator	0
Evaporation (N_2, 40°C, 60 min)	0	Occupational hazard	0	Occupational hazard	3	Occupational hazard	3
Occupational hazard	0	GC-FID	1	GC-FID	1	GC-FID	1
GC-FID	1	Waste (1-10 mL, no treatment)	6	Waste (>10 mL, no treatment)	8	Waste (>10 mL, no treatment)	8
Waste (1-10 mL, no treatment)	6						
	∑9		∑9		∑13		∑13
**Total PPs**	**29**	**Total PPs**	**29**	**Total PPs**	**76**	**Total PPs**	**55**
**Score – acceptable green analysis**	**71**	**Score – acceptable green analysis**	**71**	**Score – inadequate green analysis**	**24**	**Score – inadequate green analysis**	**45**

Table S2. Green Analytical Procedure Index (GAPI) parameters for analytical procedures (A – D) for determination of FAME in milk samples.

**Category**	**Method**			
	**A**	**B**	**C**	**D**
**Sample preparation**				
Collection (1)	Off-line	Off-line	Off-line	Off-line
Preservation (2)	Physical (low temperature)	Physical (low temperature)	Physical (low temperature)	Physical (low temperature)
Transport (3)	Required	Required	Required	Required
Storage (4)	Samples must be frozen	Samples must be frozen	Samples must be frozen	Samples must be frozen
Type of method: direct or indirect (5)	Extraction required (liquid-liquid extraction)	Extraction required (liquid-liquid extraction)	Extraction required (liquid-liquid extraction)	Extraction required (liquid-liquid extraction)
Scale of extraction (6)	Macro-extraction	Macro-extraction	Macro-extraction	Macro-extraction
Solvents/reagents used (7)	Non green solvents/reagents used	Non green solvents/reagents used	Non green solvents/reagents used	Non green solvents/reagents used
Additional treatments (8)	Derivatization	Derivatization	Derivatization	Derivatization
**Reagent and solvent**				
Amount (9)	< 10 mL	<10 mL	<10 mL	10-100 ml
Health hazard (10)	NFPA = 2 or 3 (depends on the reagent)	NFPA = 2 or 3 (depends on the reagent)	NFPA = 2 or 3 (depends on the reagent)	NFPA = 2 or 3 (depends on the reagent)
Safety hazard (11)	Highest NFPA flammability or instability score is 2 or 3	Highest NFPA flammability or instability score is 2 or 3	Highest NFPA flammability or instability score is 2 or 3	Highest NFPA flammability or instability score is 2 or 3
**Instrumentation**				
Energy (12)	≤1.5 kWh per sample	≤1.5 kWh per sample	≤1.5 kWh per sample	≤1.5 kWh per sample
Occupational hazard (13)	Emission of vapours to the atmosphere	-	-	-
Waste (14)	1-10 mL	>10 mL	>10 mL	>10 mL
Waste treatment (15)	No treatment	No treatment	No treatment	No treatment
**QUANTIFICATION**	Yes	Yes	Yes	Yes

Table S2. (*cont.*) Green Analytical Procedure Index (GAPI) parameters for analytical procedures (E – G′) for determination of FAME in milk samples.

**Category**	**Method**			
	**E**	**F**	**G**	**G′**
**Sample preparation**				
Collection (1)	Off-line	Off-line	Off-line	Off-line
Preservation (2)	Physical (low temperature)	Physical (low temperature)	Physical (low temperature)	Physical (low temperature)
Transport (3)	Required	Required	Required	Required
Storage (4)	Samples must be frozen	Samples must be frozen	Samples must be frozen	Samples must be frozen
Type of method: direct or indirect (5)	Extraction required (liquid-liquid extraction)	Extraction required (liquid-liquid extraction)	Extraction required (liquid-liquid extraction)	Extraction required (liquid-liquid extraction)
Scale of extraction (6)	Macro-extraction	Macro-extraction	Macro-extraction	Macro-extraction
Solvents/reagents used (7)	Non green solvents/reagents used	Non green solvents/reagents used	Non green solvents/reagents used	Non green solvents/reagents used
Additional treatments (8)	Derivatization	Derivatization	Derivatization	Derivatization
**Reagent and solvent**				
Amount (9)	<10 mL	<10 mL	>100 mL	10-100 ml
Health hazard (10)	NFPA = 2 or 3 (depends on the reagent)	NFPA = 2 or 3 (depends on the reagent)	NFPA = 2 or 3 (depends on the reagent)	NFPA = 2 or 3 (depends on the reagent)
Safety hazard (11)	Highest NFPA flammability or instability score is 2 or 3	Highest NFPA flammability or instability score is 2 or 3	Highest NFPA flammability or instability score is 2 or 3	Highest NFPA flammability or instability score is 2 or 3
**Instrumentation**				
Energy (12)	≤1.5 kWh per sample	≤1.5 kWh per sample	>1.5 kWh per sample	≤1.5 kWh per sample
Occupational hazard (13)	-	-	Emission of vapours to the atmosphere	Emission of vapours to the atmosphere
Waste (14)	1-10 mL	1-10 mL	>10 mL	>10 mL
Waste treatment (15)	No treatment	No treatment	No treatment	No treatment
**QUANTIFICATION**	Yes	Yes	Yes	Yes

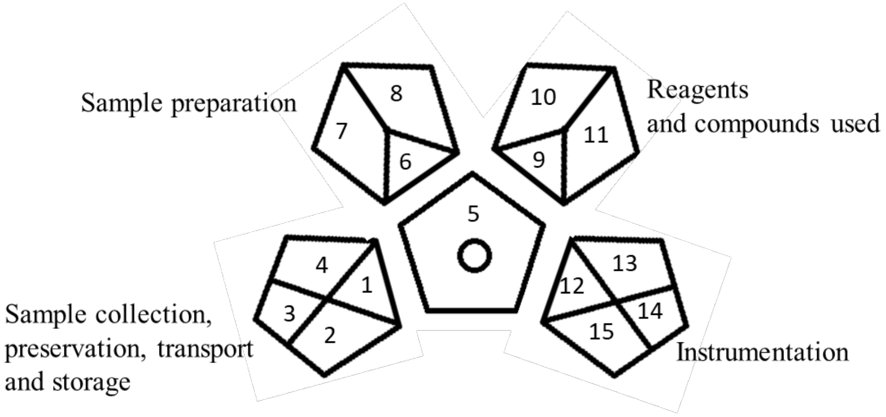

Sample preparation
1 Collection
2 Preservation
3 Transport
4 Storage
5 Type of method: direct or indirect
6 Scale of extraction
7 Solvents/reagents used
8 Additional treatments
Reagent and solvents
9 Amount
10 Health hazard
11 Safety hazard
Instrumentation
12 Energy
13 Occupational hazard
14 Waste
15 Waste treatment

Figure S2. Green Analytical Procedure Index pictogram with description.

Criteria and scores' calculation for AGREEprep [25]

The assessment criteria are based on the ten principles of green sample preparation given as below:

1. Favor in situ sample preparation
2. Use safer solvents and reagents
3. Target sustainable, reusable, and renewable materials
4. Minimize waste
5. Minimize sample, chemical and material amounts
6. Maximize sample throughput
7. Integrate steps and promote automation
8. Minimize energy consumption
9. Choose the greenest possible post-sample preparation
configuration for analysis
10. Ensure safe procedures for the operator

Reference:

[25] Wojnowski, W.; Tobiszewska, M.; Pena-Pereira, F.; Psillakis, E. AGREEprep–Analytical greenness metric for sample preparation. *Trends in Anal. Chem.* **2022**, *149*, 11655.

AGREEprep

Analytical Greenness Metric
for Sample Preparation

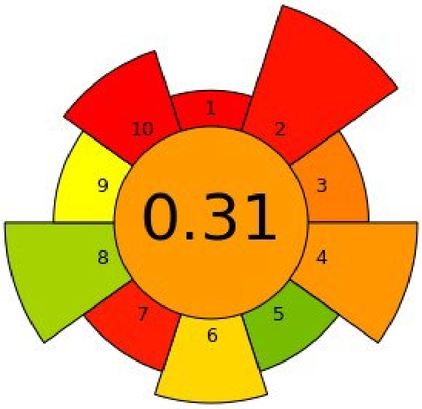

METHOD A

16/07/2022 17:58:27

#	Criterion	Score	Weight
1.	**Sample preparation placement**<br>Sample preparation placement: Ex situ	0.0	1
2.	**Hazardous materials**<br>Mass [g] or volume [mL] of problematic materials: 7.4	0.04	5
3.	**Sustainability and renewability of materials**<br>25-50% of reagents and materials are sustainable or renewable	0.25	2
4.	**Waste**<br>Mass [g] or volume [mL] of waste: 7.9	0.3	4
5.	**Size economy of the sample**<br>Mass [g] or volume [mL] of the sample: 0.5	0.77	2
6.	**Sample throughput**<br>Hourly sample throughput: 6	0.42	3
7.	**Integration and automation**<br>No. of sample prep. steps: 5 steps; degree if automation: Manual systems	0.06	2
8.	**Energy consumption**	0.68	4

	Approximate energy consumption per analysis [W]: 35		

9.	**Post-sample preparation configuration for analysis**<br>GC with non-MS detection, atomic absorption spectroscopy, capillary electrophoresis, etc.	0.5	2

10.	**Operator's safety**<br>No. of distinct hazards: 4 or more hazards	0.0	3

AGREEprep

Analytical Greenness Metric
for Sample Preparation

METHOD B

16/07/2022 18:22:01

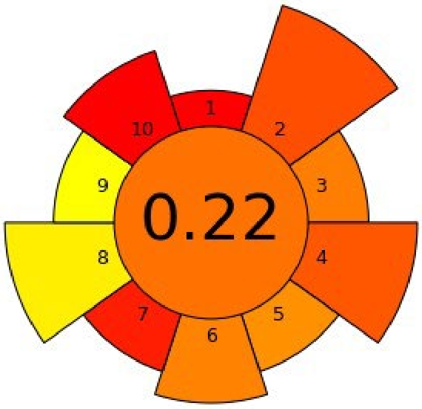

#	Criterion	Score	Weight
1.	**Sample preparation placement**<br>Sample preparation placement: Ex situ	0.0	1
2.	**Hazardous materials**<br>Mass [g] or volume [mL] of problematic materials: 3.4	0.16	5
3.	**Sustainability and renewability of materials**<br>25-50% of reagents and materials are sustainable or renewable	0.25	2
4.	**Waste**<br>Mass [g] or volume [mL] of waste: 17.4	0.17	4
5.	**Size economy of the sample**<br>Mass [g] or volume [mL] of the sample: 14	0.28	2
6.	**Sample throughput**<br>Hourly sample throughput: 3	0.26	3
7.	**Integration and automation**<br>No. of sample prep. steps: 5 steps; degree if automation: Manual systems	0.06	2
8.	**Energy consumption**	0.47	4

	Approximate energy consumption per analysis [W]: 80		

9.	**Post-sample preparation configuration for analysis**	0.5	2
	GC with non-MS detection, atomic absorption spectroscopy, capillary electrophoresis, etc.		

10.	**Operator's safety**	0.0	3
	No. of distinct hazards: 4 or more hazards		

AGREEprep

Analytical Greenness Metric
for Sample Preparation

METHOD C

16/07/2022 18:26:30

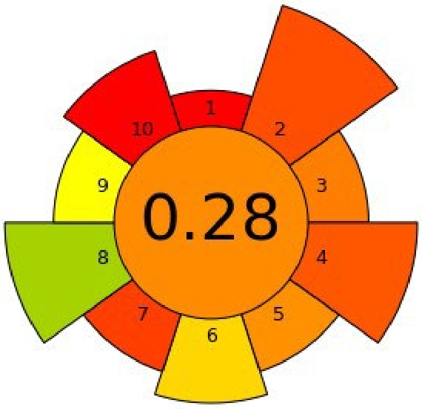

#	Criterion	Score	Weight
1.	**Sample preparation placement**<br>Sample preparation placement: Ex situ	0.0	1
2.	**Hazardous materials**<br>Mass [g] or volume [mL] of problematic materials: 3.4	0.16	5
3.	**Sustainability and renewability of materials**<br>25-50% of reagents and materials are sustainable or renewable	0.25	2
4.	**Waste**<br>Mass [g] or volume [mL] of waste: 17.4	0.17	4
5.	**Size economy of the sample**<br>Mass [g] or volume [mL] of the sample: 14	0.28	2
6.	**Sample throughput**<br>Hourly sample throughput: 6	0.42	3
7.	**Integration and automation**<br>No. of sample prep. steps: 4 steps; degree if automation: Manual systems	0.12	2
8.	**Energy consumption**	0.68	4

	Approximate energy consumption per analysis [W]: 35		

9.	**Post-sample preparation configuration for analysis**	0.5	2
	GC with non-MS detection, atomic absorption spectroscopy, capillary electrophoresis, etc.		

10.	**Operator's safety**	0.0	3
	No. of distinct hazards: 4 or more hazards		

AGREEprep

Analytical Greenness Metric
for Sample Preparation

METHOD D

16/07/2022 18:26:30

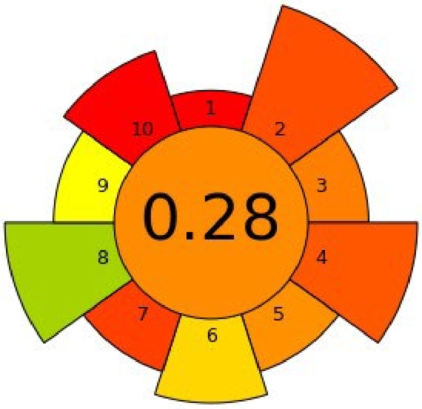

#	Criterion	Score	Weight
1.	**Sample preparation placement**<br>Sample preparation placement: Ex situ	0.0	1
2.	**Hazardous materials**<br>Mass [g] or volume [mL] of problematic materials: 3.4	0.16	5
3.	**Sustainability and renewability of materials**<br>25-50% of reagents and materials are sustainable or renewable	0.25	2
4.	**Waste**<br>Mass [g] or volume [mL] of waste: 17.4	0.17	4
5.	**Size economy of the sample**<br>Mass [g] or volume [mL] of the sample: 14	0.28	2
6.	**Sample throughput**<br>Hourly sample throughput: 6	0.42	3
7.	**Integration and automation**<br>No. of sample prep. steps: 4 steps; degree if automation: Manual systems	0.12	2
8.	**Energy consumption**	0.68	4

	Approximate energy consumption per analysis [W]: 35		

9.	**Post-sample preparation configuration for analysis**	0.5	2
	GC with non-MS detection, atomic absorption spectroscopy, capillary electrophoresis, etc.		

10.	**Operator's safety**	0.0	3
	No. of distinct hazards: 4 or more hazards		

AGREEprep

Analytical Greenness Metric
for Sample Preparation

METHOD E

16/07/2022 18:47:36

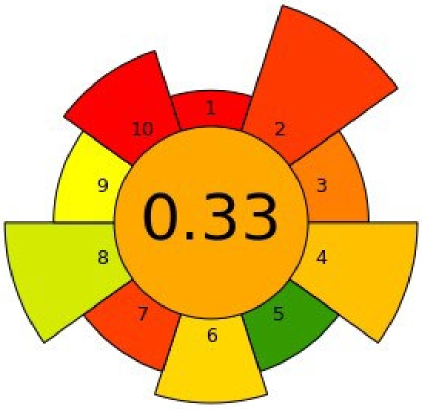

#	Criterion	Score	Weight
1.	**Sample preparation placement**<br>Sample preparation placement: Ex situ	0.0	1
2.	**Hazardous materials**<br>Mass [g] or volume [mL] of problematic materials: 4.5	0.12	5
3.	**Sustainability and renewability of materials**<br>25-50% of reagents and materials are sustainable or renewable	0.25	2
4.	**Waste**<br>Mass [g] or volume [mL] of waste: 4.7	0.38	4
5.	**Size economy of the sample**<br>Mass [g] or volume [mL] of the sample: 0.2	0.9	2
6.	**Sample throughput**<br>Hourly sample throughput: 6	0.42	3
7.	**Integration and automation**<br>No. of sample prep. steps: 4 steps; degree if automation: Manual systems	0.12	2
8.	**Energy consumption**	0.59	4

	Approximate energy consumption per analysis [W]: 50		

9.	**Post-sample preparation configuration for analysis**	0.5	2
	GC with non-MS detection, atomic absorption spectroscopy, capillary electrophoresis, etc.		

10.	**Operator's safety**	0.0	3
	No. of distinct hazards: 4 or more hazards		

AGREEprep

Analytical Greenness Metric
for Sample Preparation

METHOD F

16/07/2022 18:54:36

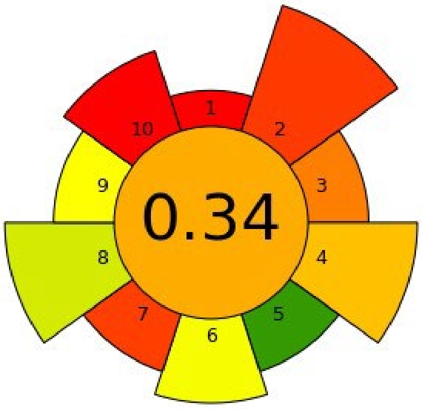

#	Criterion	Score	Weight
1.	**Sample preparation placement**<br>Sample preparation placement: Ex situ	0.0	1
2.	**Hazardous materials**<br>Mass [g] or volume [mL] of problematic materials: 4.5	0.12	5
3.	**Sustainability and renewability of materials**<br>25-50% of reagents and materials are sustainable or renewable	0.25	2
4.	**Waste**<br>Mass [g] or volume [mL] of waste: 4.7	0.38	4
5.	**Size economy of the sample**<br>Mass [g] or volume [mL] of the sample: 0.2	0.9	2
6.	**Sample throughput**<br>Hourly sample throughput: 9	0.52	3
7.	**Integration and automation**<br>No. of sample prep. steps: 4 steps; degree if automation: Manual systems	0.12	2
8.	**Energy consumption**	0.59	4

	Approximate energy consumption per analysis [W]: 50		

9.	**Post-sample preparation configuration for analysis**	0.5	2
	GC with non-MS detection, atomic absorption spectroscopy, capillary electrophoresis, etc.		

10.	**Operator's safety**	0.0	3
	No. of distinct hazards: 4 or more hazards		

AGREEprep

Analytical Greenness Metric
for Sample Preparation

METHOD G

20/07/2022 11:13:08

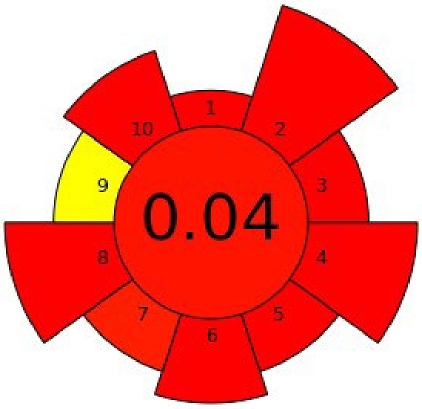

#	Criterion	Score	Weight
1.	**Sample preparation placement**<br>Sample preparation placement: Ex situ	0.0	1
2.	**Hazardous materials**<br>Mass [g] or volume [mL] of problematic materials: 352	0.0	5
3.	**Sustainability and renewability of materials**<br>< 25% of reagents and materials are sustainable or renewable and can only be used once	0.0	2
4.	**Waste**<br>Mass [g] or volume [mL] of waste: 750	0.0	4
5.	**Size economy of the sample**<br>Mass [g] or volume [mL] of the sample: 100	0.0	2
6.	**Sample throughput**<br>Hourly sample throughput: 0.7	0.0	3
7.	**Integration and automation**<br>No. of sample prep. steps: 5 steps; degree if automation: Manual systems	0.06	2
8.	**Energy consumption**	0.0	4

	Approximate energy consumption per analysis [W]: 1640		

9.	**Post-sample preparation configuration for analysis**	0.5	2
	GC with non-MS detection, atomic absorption spectroscopy, capillary electrophoresis, etc.		

10.	**Operator's safety**	0.0	3
	No. of distinct hazards: 4 or more hazards		

AGREEprep

Analytical Greenness Metric
for Sample Preparation

METHOD G'

20/07/2022 11:16:41

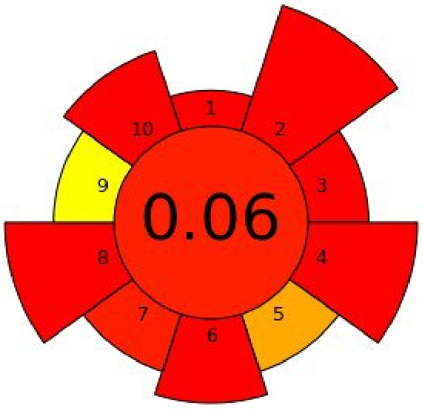

#	Criterion	Score	Weight
1.	**Sample preparation placement**<br>Sample preparation placement: Ex situ	0.0	1
2.	**Hazardous materials**<br>Mass [g] or volume [mL] of problematic materials: 35	0.0	5
3.	**Sustainability and renewability of materials**<br>< 25% of reagents and materials are sustainable or renewable and can only be used once	0.0	2
4.	**Waste**<br>Mass [g] or volume [mL] of waste: 75	0.0	4
5.	**Size economy of the sample**<br>Mass [g] or volume [mL] of the sample: 10	0.33	2
6.	**Sample throughput**<br>Hourly sample throughput: 1	0.0	3
7.	**Integration and automation**<br>No. of sample prep. steps: 5 steps; degree if automation: Manual systems	0.06	2
8.	**Energy consumption**	0.0	4

	Approximate energy consumption per analysis [W]: 1000		

9.	**Post-sample preparation configuration for analysis**	0.5	2
	GC with non-MS detection, atomic absorption spectroscopy, capillary electrophoresis, etc.		

10.	**Operator's safety**	0.0	3
	No. of distinct hazards: 4 or more hazards		
